# ﻿The history and typification of *Liliumbrownii* A.Lemoinier (Liliaceae)

**DOI:** 10.3897/phytokeys.195.81755

**Published:** 2022-05-05

**Authors:** James A. Compton

**Affiliations:** 1 Spilsbury Farm, Tisbury, SP3 6RU, UK Unaffiliated Tisbury United Kingdom

**Keywords:** Brown nursery, China, Horticultural Society Kerr, Kew, *Liliumbrownii*, *L.japonicum*, nomenclature, Reeves, typification

## Abstract

The Chinese *Liliumbrownii* has been much confused with the Japanese endemic species *Liliumjaponicum*. In this paper, it is shown that *L.brownii* was introduced to England at least four times between 1804 and 1819. The history of *L.brownii* is fully discussed and its taxonomy, nomenclature and relationships are examined. A neotype is designated for the name, its correct botanical authority is given and the correct place of its publication is provided. Lectotypes are also provided for the names *Liliumaduncum* Stapf, *Liliumaustrale* Stapf, *Liliumodorum* Planch., Liliumbrowniivar.colchesteri E.H.Wilson and Liliumbrowniivar.ferum Stapf.

## ﻿Introduction

Nine species names have been given to Chinese species of the genus *Lilium* L. that have infundibuliform or trumpet-shaped flowers (Liang and Tamura 2008). The first of these to be formally described with a Latin name, is currently accepted as *Liliumbrownii* F.E.Brown ex Miellez in 1841. This species with a widespread distribution across central and southern China has been known to the Chinese as an important medicinal and culinary plant under the name “pae hup” in Cantonese or “bai he” in Mandarin from as early as the Tang dynasty (618–907). This species is known today by the very similar sounding Cantonese name of Pak Hup 百合.

Since the species was first introduced to England from Canton [Guangzhou] China as an ornamental in 1804, it has been persistently confused with the Japanese endemic species *Liliumjaponicum* Thunb. ex Houtt. and was, on its arrival, initially given that name (W.T.Aiton 1811: 240). This initial misidentification was a problem which persisted well into the late 19^th^ century (e.g. Baker 1875: 230). The two species share some morphological similarities, but can readily be distinguished (see confusion with *L.japonicum* below). To add to this, several illustrations have been made of *Liliumbrownii* by Japanese artists in Japan owing to the introduction of the species to that country and its subsequent cultivation there since at least the 16^th^ century (Okubo et al. 2012).

*Liliumbrownii* has also been confused with other trumpet-flowered species, in particular with *L.longiflorum* Thunb., a species native to Japan, the Ryukyu Islands and along the northern coast of Taiwan. The two species both have white, trumpet-shaped flowers, but *L.longiflorum* has no reddish-brown colouration on the outside of the corolla and the anthers carry bright yellow not reddish-brown pollen.

The French missionary botanist Julien Cavalerie’s uncertainty of the distinctions between these Chinese trumpet-flowered lilies is exemplified by his description of *L.sulphureum* Baker ex Hook.f. (Hooker 1892: 351) under the name *L.brownii*. This misidentification was yet further exacerbated by his description of *L.brownii* immediately afterwards under the name *L.longiflorum* (Cavalerie 1911: 245). *Liliumbrownii* has even been considered to be of hybrid origin, albeit without any evidence to support that suggestion (e.g. Franchet 1892: 312; R.Wallace 1932: 51). When Louis van Houtte made a comparison between Liliumlongiflorumvar.suaveolens and what he was calling *L.japonicum*, he specifically mentioned that his “*L.japonicum*” had violet-purple internal colouring and dark chocolate brown pollen (Van Houtte 1833: 182). He may have been referring to a hybrid with *L.brownii* as a putative parent.

At no stage in its botanical history has a type been allocated to the species. The liliophile Kew botanist John Gilbert Baker segregated L.browniivar.viridulum from (by implication) var. brownii on the shorter, wider, more oblanceolate leaves and paler greenish colouration on the outside of the corollas with less pronounced claret markings (Baker 1885: 131). Baker’s statement “The leaves are much broader and shorter than in the type” is almost certainly intended to refer to what he regarded as the typical variety of *L.brownii*, a point further indicated by his citation of (Mielle) [i.e. Miellez] as the author of the name and which was accompanied by a reference to the description and illustration in *Flore des Serres* by Charles Lemaire. The latter portrays a plant with linear-lanceolate leaves and a flower with reddish markings on the outside of the perianth (Lemaire 1845: t. 47). These references, however, do not constitute typification of the species. William Stearn regarded what he called L.browniivar.brownii as being based on L.japonicumvar.brownii (Spae) Baker (Baker 1871: 709); however, he again did not indicate any type specimen or illustration (Stearn 1948: 5).

All authors prior to this paper have followed the Belgian botanist Dieudonné Spae (1819–1858) by wrongly attributing the name of the plants from which the species originated to F. E. Brown of the Slough nursery near Windsor, England (Spae 1845: 438; 1847: 12). It is shown here that, although the Slough nursery was indeed the source of Spae's plants, no such member of the Brown family with those initials was ever involved therein. It is shown here too that Miellez, who was also credited with authorship of the name, never validly described the species.

## ﻿William Kerr’s introductions from Canton to Kew

The bulbs of the as yet unnamed *Liliumbrownii* were first unloaded at the wharves of the East India Company’s dockyard, Blackwall, London on 14 August 1804 (Hardy 1811: 222). They were part of a consignment of plants collected by William Kerr (1779–1814), a Scottish gardener at the Royal Garden at Kew who was sent to China’s southern port of Canton in 1803 by Sir Joseph Banks, special advisor to King George III. His mission was to remain in Canton specifically to collect plants, which he did until 1812 (Goodman and Jarvis 2017: 269). Kerr’s sending of his shipments from Canton to Kew ceased in 1810. The bulbs that were sent with his first shipment were put in a box at Canton on the 1200 ton Honourable East India Company’s ship [HEICS] “Henry Addington”, commanded by Captain John Kirkpatrick (1766–1816) for the long sea journey back to England. The Henry Addington set sail from Canton leaving the anchorage below the Second Bar Island, 12 miles (ca. 19 km) south of Whampoa Island [Pazhou island], Canton on 1 February 1804 for its homeward bound journey. This took the ship around the Cape of Africa with a stop off at the South Atlantic Ocean island of Saint Helena which was then under the governorship of the East India Company.

It is remarkable that the ship with the bulbs on board survived that journey. The “Henry Addington” was involved in the Battle of Pulo Aura [Pulau Aur] between the British and the French following the collapse of the Treaty of Amiens in 1803 and the reconvening of the Napoleonic Wars. The ship was part of a large convoy of British merchant ships that set off from China and sailed through the Straits of Malacca under the command of Sir Nathaniel Dance, commodore of the EIC fleet. This convoy encountered four roving French warships and a Dutch brig under the command of the French Contre-Admiral Charles comte de Linois on 15 February 1804 who, believing it to be a fleet of British warships, left the scene after only a skirmish (Hardy 1811: appendix 123).

The first written record of this shipment of plants was in the list put together by William Kerr in his “*Memorandum of Plants, Seeds & c. sent from China to the Royal Gardens, Kew*” which is now conserved in the library of the School of Oriental and African Studies (SOAS) in London (Kerr 1804). Included as the first part of Kerr’s journal is a “*Catalogue of plants procured at Canton, China and sent to England on board the ship Henery Addington (sic) in a greenhouse or plant cabin prepared for the purpose. This ship with the whole China Fleet of the season sailed from the Second Bar Canton River Feb. 1^st^ 1804*” (Kerr 1804: fol. 1). This “Memorandum” recorded the first of about a dozen shipments of plants that Kerr sent back to Kew from 1804 until 1810 (Kew Record Book 1804–1826).

William Kerr did not elucidate how or from where he had acquired the plants that he had put on board the EIC ship. During his time in Canton after his arrival in late 1803, he frequently visited the garden nurseries at Fa-tee or Fati [Huadi] “flowery land” across the Pearl [Zhujiang] River and a little upstream from where he was compelled to reside and spend the majority of his time in the British factory (Fan 2003: 71). This building set back from, but facing the river was one of 17 elaborately-fronted foreign offices and warehouses all known as the “factories” along the river at Xiguan (Livingstone 1819: 126). From 1757, the Qianlong Emperor (1711–1799) closed China to all foreign trade, except that which was permitted from the ports of Canton and Macao. This restriction continued until the treaties that emerged as a result of the Anglo-Chinese opium wars (1839–1842). When Kerr arrived in China, trade with the Chinese within Canton by foreigners was severely restricted to within these factories and to the houses of the Chinese “Hong” merchants and was only permitted to take place during the winter months i.e. between October and March (Compton 2015: 265). This explains why it was that, as Kerr himself stated, he did not see these lilies when they came into flower in June or July. In his “Memorandum”, Kerr stated that all the plants on board the HEICS “Henry Addington” during the journey to England were carefully tended by his friend Mr. Allen (Kerr 1804 fol. 78). Kerr had clearly met and befriended John Allen, a Derbyshire miner who was passing through Canton on his way back to England from Australia (Kilpatrick 2007: 168).

Kerr’s entry for number nine on his list included the Chinese name “Pae-hup-fa” with “fa” meaning flower in Cantonese and, next to this entry, he placed four crosses (“xxxx”). Kerr does not indicate what these four crosses symbolised, but it would have been the level of desirability according to the code for desiderata designed by Sir Joseph Banks. These symbols relate to the list of Chinese plants and their corresponding illustrations in “The Book of Chinese Plants” which he had been lent by Banks (Goodman and Jarvis 2017: 266). Thus four crosses next to the name of a plant meant that it was an unknown plant of high desirability, reducing in the value of its desirability down to one cross x = known, but not seen living (Goodman and Jarvis 2017: 266). Kerr mentions in his “Memorandum” for his entry number one: “*T’hoi tong-fa* Begonia *fig. 4 xxx in the Chinese Book of Drawings brought out by Mr Lance*”. This book was a quarto book of Chinese plant illustrations which was most probably based on others undertaken previously by Chinese artists for John Bradby Blake’s visits to Canton. Blake was a supercargo [merchant] for the EIC from 1766 until his death in Canton in 1773 (Goodman and Jarvis 2017: 252, 266). The illustrated book was designed to aid in the identification of the Chinese plants so that those collected by Kerr were not duplicated.

Kerr also included in his “Memorandum” a square symbol (“□”) which meant that the plants were placed in a wooden box. The number of squares placed next to a plant’s name indicated the number of boxes loaded on board ship (Kerr 1804: fol. 1). Next to number nine in the “Memorandum”, Kerr added the script:

“*9. Pae-hup-fa fig. 36 xxxx This is a bulbous rooted plant. The bulb resembles that of*Liliumbulbiferum. *I have neither seen the flowers nor leaves. Used in medicine as well as for ornament □ 1.*”

Kerr’s mention of “fig. 36” most probably refers to an illustration of this plant in “The Book of Chinese Plants” brought to him by Mr. David Lance who had been tasked to hold overall responsibility for Kerr’s welfare in Canton. Lance, a friend of Sir Joseph Banks and a senior supercargo in Canton, had travelled out from England with Kerr along with the ship’s surgeon and keen botanist John Livingstone on the HEICS “Coutts”, commanded by Captain Robert Torin (Kilpatrick 2007: 165). The “Coutts” left The Downs in Kent on 6 May 1803 and did not arrive in Whampoa, Canton, until 1 October 1803 (Hardy 1811: 228). The ship survived a disastrous typhoon which destroyed both of the ship’s masts and caused the loss of the anchors overboard, necessitating the ship to be towed into Canton (Kerr 1804 fol. 75). The “Book of Chinese Plants” must have been given into Lance’s safe-keeping by Sir Joseph Banks and is now missing. There is also another entry on Kerr’s “Memorandum” list: number 19 “Kun-tan” xxx (= unknown and desirable) and the statement: “Lilium? *I have not yet seen the leaves or flowers, the bulbs resemble those of*Liliumcandidum”.

These were the only lilies that Kerr included in this, his first list delivered to the Royal Garden at Kew. Kerr’s description of his number nine “having bulbs resembling *Liliumbulbiferum*” equates to the whitish bulbs of *L.brownii*.

In the Kew Record Book (1804–1826), which holds records of all the plants arriving into the Royal Gardens, there are a number of similar entries referring to the various dispatches of Kerr’s plants from Canton. These entries are carefully cross-referenced by Kerr to correspond to the numbered plants in his *Memorandum* and to the illustrations in the “Book of Chinese Plants”. On the first folio of the Kew Record Book, Kerr added some additional information regarding this first collection of his plants: “*As far as number 62 are all cultivated plants either for ornament or use*”. Later he added: “*From number 62 are wild plants collected in Danes Island*”. The significance of this statement is that his number nine “Pae-hup-fa” was a cultivated and not a wild plant. Danes Island [Changzhou Island] next to Whampoa held a Danish cemetary. In Kew Record Book 1804: fol. 5, the full entry for number nine states:

“*9. Pae-hup-fa fig. 36 xxxx A liliaceous and bulbous rooted plant, the roots resemble those of*Liliumcandidum. *I have not yet seen either flowers or leaves. It is a very scarce plant here and is originally from Nan-Kin, the roots are used in medicine*”

It should be noted that Kerr’s switching of the resemblance of the bulbs from *L.bulbiferum* L. in his “Memorandum” to *L.candidum* L. in the Kew Record Book is of little significance as the bulbs of both species are very similar. His reference to Nan-kin [Nanjing, Jiangsu Province] is unknown, but may refer to his belief that the lily had a more northern wild distribution.

Later in the Kew Record Book (1804–1826), there is a second reference to Kerr sending more bulbs of *Liliumbrownii*. Kerr dispatched plants “*in the plant cabin aboard the HEICS Hope with Captain Pendergrass*”. These were sent back from Canton on 23 February 1806 (Kew Record Book 1806: 47). The entry simply states: “*Number 27 Pa-hup* Lillium *sp. (sic.) 1* [box]”. The 1200 ton *Hope* arrived back in London on 7 September 1806 (Hardy 1811: 246).

## ﻿First description of the Chinese trumpet-lily

The superintendant of the Royal Garden at Kew, William Townsend Aiton (1766–1849) was the first to describe the new Chinese lily as *Liliumjaponicum* (Aiton 1811: 240). His description was based on the lily’s first flowering in cultivation at Kew and appeared in the second volume of the second edition of Hortus Kewensis, the catalogue of the plants cultivated in the garden. Aiton called it the “White Japan Lily” stating that it had come from China in 1804, courtesy of William Kerr on the HEICS*Henry Addington* under Captain Kirkpatrick. The second of Kerr’s Chinese lilies, i.e. his “kun-tan”, also flowered and was described under the name *L.tigrinum* Ker Gawl. (W.T.Aiton 1811: 241). This was almost certainly what is now recognised as *L.lancifolium* Thunb. Aiton added that this species had also been sent by Kerr with Captain Kirkpatrick to Kew in 1804.

**Figure 1.**
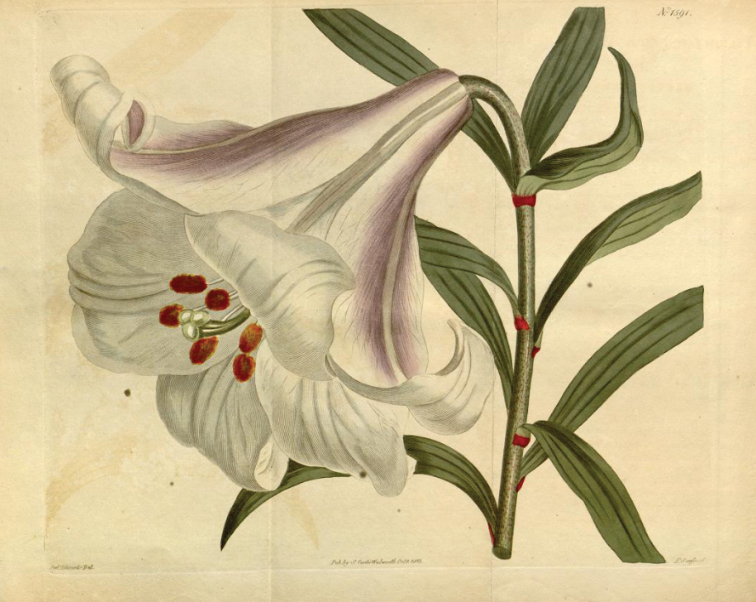
A good representation of Liliumbrowniivar.brownii A.Lemoinier, based on William Kerr’s original collection published in “Curtis’s Botanical Magazine” 38 t. 1591 (1813) and labelled as *Liliumjaponicum*.

Kerr’s new lily introduction was once again fully described under the name *Liliumjaponicum* by John Bellenden Ker-Gawler along with a coloured illustration by Sydenham Edwards (see Fig. 1) in Curtis’s Botanical Magazine, volume 38 (Ker-Gawler 1813: t. 1591). Gawler added to the confusion by stating that the lily was native to both China and Japan. He cited *L.japonicum* Thunb. and thanked William Townsend Aiton for being able to depict the plant which had flowered for the first time at Kew in July 1812, although Aiton must have described it flowering before 1811 (Aiton 1811: 240). There is no doubt that this is *L.brownii*.

The Belgian nobleman and politician François de Cannart d’Hamale wrote a literary appraisal of all the previously-published works on the genus *Lilium* up to the 1860s (Cannart d’Hamale 1870). In this work, he stated that the lis du Japon (*L.japonicum* Thunb.) had taken some years to arrive in France following its introduction to Europe in 1804 by the directors of the [British] East India Company courtesy of Captain Kirkpatrick and that, in France, it had first flowered in the garden of Monsieur Dumont at Courset near Boulogne in 1809 (Cannart d’Hamale 1870: 394). This first flowering in France was also undoubtedly of *L.brownii*, based on that description and, if correct, had come into flower two years before the plants had first flowered at Kew.

## ﻿Confusion with *Liliumjaponicum* Thunb. ex Houtt.

*Liliumjaponicum*, the Japanese bamboo lily or sasa-yuri was first validly, but rather poorly described by the Dutch botanist Maarten Houttuyn, accompanied by a far from convincing illustration of a single unopened trumpet-shaped flower (Houttuyn 1780: 245 t. 82, f. 2). According to Houttuyn, the depiction and description of the new lily was based on one of Thunberg’s collections from Japan in 1775 and 1776 and Houttuyn stated that Thunberg called it the Japanese Lily. Thankfully, four years later, Thunberg himself added a more comprehensive description that diagnostically identified this species as having petiolate, lanceolate leaves and a campanulate white flower (Thunberg 1784: 133). There is an original specimen of this species conserved amongst the Thunberg collections in Uppsala (UPS-THUNB 8137, catalogue number V-008137 and another in Geneva G-00818143).

*Liliumjaponicum* which is endemic to the southern parts of the Japanese islands does occasionally produce white flowers, but these are predominantly of a beautiful pale pinkish colour which would not have shown in dried herbarium material. The species consists of three accepted varieties: Liliumjaponicumvar.japonicum with leaves 5–10 cm long, with a pale rose-coloured infunduliform corolla with tepals 12–15 cm long; var. abeanum (Honda) Kitam., Acta Phytotax. Geobot. 14: 121 (1952) with corollas white or light pink 5–7 cm long and var. angustifolium (Makino) Makino, J. Jap. Bot. 1(5): 16 (1917), with pink corollas and leaves 11–20 cm long (Hayashi 2016: 117). Liliumjaponicumvar.japonicum occurs in damp woods of the central and western parts of Honshu, Kyushu and Shikoku; Liliumjaponicumvar.abeanum occurs only in Tokushima Prefecture on Shikoku Island and Liliumjaponicumvar.angustifolium occurs only in the wet forests of the Kii Peninsula in southern Honshu (Hayashi 2016: 117).

The lack of diagnostic characters in the original protologue undoubtedly muddied distinctions between Thunberg’s *L.japonicum* and the arrival of *L.brownii*. *Liliumjaponicum* frequently also has brown pollen, thus the initial confusion with *L.brownii* is more understandable. The petiolate and lanceolate tapering leaves of the endemic Japanese lily whose delicate flowers are carried on a narrow stem are characteristic and show its superficial resemblance to bamboo; hence, its Japanese name. This contrasts with the more robust Chinese species with thicker lanceolate or oblanceolate leaves, absence of petioles and whose white flowers are purplish (rarely greenish) tinted only on the outside of the perianth and are especially dark streaked along the mid-rib of each tepal. In addition the margins of the nectary furrows on the perianth segments of *L.japonicum* are consistently glabrous, whereas those of *L.brownii* are frequently densely papillose. In addition, their native habitats do not overlap; *L.brownii* is endemic to China, whereas *L.japonicum* is restricted to the Japanese islands.

The description of *Liliumjaponicum* by the French botanist Jean Poiret seems to refer to the true Japanese species, not to the Chinese species under that name, as he described petiolate leaves and he failed to mention the dark red colouration on the outside of the flower to be found on *L.brownii* (Poiret 1813: 456). There is a question regarding the basis of his description - did he describe plants that he had seen in cultivation or, more likely, was he merely repeating the description of it provided by Thunberg? Murmurings of doubt as to the identity of this species in cultivation seem to have occurred a year later in the Supplément to volume seven of the second edition of Dumont de Courset’s “Le botaniste cultivateur” (Dumont de Courset 1814: 54). Dumont stated under the title “*Autres espèces cultivées*: *1. Lis de Japon*, Liliumjaponicum*Thunb*. *An*L.sinense*Hortul*.? *An*L.concolor? Smith. *Feuillesradicales*, *longues*, *lancéolées*, *pétiolées*, *acuminées*, *très-entières*, *glabres*, *bordées etc*.” [basal leaves long, lanceolate, petiolate, apices acuminate, margins completely entire, glabrous, veined etc.] and later “*Le Japon*, *où l’on cultive ce lis pour sa beauté*. *Fleurit en Juin*” (Dumont de Courset 1814: 54–55). The latter statement “in Japan where this lily is cultivated for its beauty” does not mention it as being a native of that country, yet might refer to either species.

Japan at that time was under strict Sakoku (locked in) without access to trade with all foreign nations, except with the Dutch until the opening of the country in the late 1850s. The Dutch were permitted to trade with the Japanese only from their little island of Dejima in Nagasaki Bay, but were in political upheavel at this time as a result of conflict with the British. The Kingdom of Holland, as a client state of the French during the Napoleonic wars (1803–1815), were the principal power in the Dutch East Indies. The presence of Dutch ships in the western Pacific Ocean inevitably involved the Dutch coming into confrontation with the British who took the Javanese city of Batavia [Jakarta] in 1811. The British did not return the island of Java to the Dutch until 1814 and consequently trade with the Dutch from Japan had more or less , then to Europe and ground to a standstill. Hitherto, all trade by the Dutch from Japan went first to Java which included the transportation of all Japanese plants. Is it, therefore, too far a leap to suggest that most (if not all) of the lilies cultivated at that time in Europe under the name *L.japonicum* were in fact *L.brownii* (An *L.sinense* Hortul.? of Dumont de Courset 1814: 54) and not the delicate Japanese species described by Houttuyn and Thunberg? Certainly the illustrations of plants in Europe named *L.japonicum* at that time all appear to represent *L.brownii*.

The question of the misidentification and misapplication of the name *L.japonicum* to *L.brownii* and the uncertainty surrounding the identity of the true *L.japonicum* and its synonym *L.krameri* Hook.f., Bot. Mag. 99 t. 6058 (1873) was set to continue as subsequent introductions of both species arrived from China and Japan respectively throughout the later 19^th^ Century (e.g. A.Wallace 1875: 292; Elwes 1877: t. 8; A.Wallace 1878: 505; Krelage 1878: 541).

## ﻿John Reeves’s introductions from Canton to the Horticultural Society

Two more introductions of the lily as *L.japonicum* were reported to have arrived in London from China in 1819 (Loddiges 1820: t. 438; Brookes 1822: 551). The lily from one of these introductions was described and painted by George Loddiges and engraved by George Cooke in “Loddiges Botanical Cabinet” (Loddiges 1820: t. 438). Loddiges mentioned that the plant that grew in China and Japan had been introduced by the Horticultural Society of London. This Society was founded in 1804 with Sir Joseph Banks as one of its founding members and it eventually became the Royal Horticultural Society after 1859. Loddiges did not specify the precise origin of the painted plant nor the exact date of its arrival in England, but his praise of Joseph Sabine for the distribution of plants from the Society indicated that it was Sabine who must have been the provider of this plant to his famous Hackney nursery.

The source of the lily in China would have been John Reeves (1774–1856) who was then the EIC Assistant Inspector of teas in Canton from 1812 to 1826, thence Chief Inspector to 1831. Reeves had been in China since 1812 following the loss of his wife Sarah Russell in 1810. In May 1816, Reeves returned to England to resuscitate his health from the subtropical heat and to marry his fiancée Isabella Andrew as his second wife (Bailey 2019: 83). For the next year, he was to work in India House for the EIC, returning to his duties in China in 1817 (for additional information on Reeves, see Bailey 2019). It was part of Sir Joseph Banks’s request to Reeves as it had earlier been to Kerr to have Chinese plants illustrated. In this case, not for Kew, but for the Horticultural Society, in order for the Society to see and make a judgement on the merits of the plants prior to granting approval for their introduction. During his visit home, Reeves must have met Joseph Sabine, the Secretary of the Society to discuss the idea of commissioning Chinese artists to undertake the illustrations. The Horticultural Society’s Council Minutes for 18 February 1817 recorded: “*That the proposal of John Reeves esq. to send plants and drawings from China for the use of the Society, be accepted with thanks and that the Secretary do offer to Mr. Reeves the advance of such sums as he may require towards the cost of the same*”. On 1 April 1817, the Council Minutes simply stated “£*25 to Mr. Reeves for executing the said instructions*”.

In 1817, the Horticultural Society did not possess a garden in which to put any plants arriving from abroad. Council Minutes 17 February 1818 reveal, however, that the Society was negotiating with a Mr. Sutton for the lease of ground for a garden in Kensington and had agreed to employ Charles Strachan as gardener. Council Minutes 29 April 1818 indicated the arrival of Chinese plants and their current lack of garden facilities: “*The Secretary reported that he had received advice of the arrival of some plants from China for the Society which Mr. Lee of Hammersmith had offered to take charge of for the Society, which offer was accepted with thanks*”. The famous Hammersmith nursery firm of Lee and Kennedy founded ca. 1745 was by this time under the management of the younger James Lee (1754–1824) and his partner John Kennedy (1759–1842).

On Tuesday 16 June 1818, the Council Minutes relate that: “*Mr Reeves’s expenditure thus far on plants and drawings amounted to £25 and that an advance of a further £25 was to be made for next season*”.

On 7 July 1818, the Council Minutes provided a comprehensive description of the arrival of two shipments of Chinese plants for the Society from Mr. Reeves in Canton and that these were sent to Mr. William Anderson, curator of the Botanic Garden in Chelsea [now Chelsea Physic Garden]. John Reeves had entrusted their care during the long journey from China into the hands of two ship’s captains; Captain Archibald Hamilton of the 1242 ton HEICS “Bombay” and Captain Charles Mortlock of the 1507 ton HEICS “Lowther Castle”. Council also thanked Mr. David Maclean of the Customs House for his care of the plants and drawings on their arrival in London. The fifth voyage of HEICS “Bombay” left the Second Bar, Canton on 22 November 1817 and arrived at Long Reach, Gravesend on 20 May 1818. The fourth voyage of HEICS “Lowther Castle” left the Second Bar on 19 December 1817 and arrived at The Downs on 2 June 1818 (Hardy 1820: 340). The two shipments, therefore, arrived within a fortnight of each other.

The same Minutes on 7 July 1818 stated that “*29 Chinese Drawings arrived having been directed by Mr. Reeves and these were examined and approved by Council*”.

The RHS Lindley Library has two paintings of *Liliumbrownii* under the name *L.japonicum* undertaken in China by Chinese artists working for John Reeves on behalf of the Society. These are catalogued as A/REE/SmV5/5 (small volume page 5) and A/REE/SmV5/114 (small volume page 114) and, due to their time of flowering i.e. June-July, would have been undertaken during the summer in the Company Factory House in Macao. There is no additional data on the arrival in England of the first of these, but it may have coincided with the introduction of bulbs of the Chinese species under the name *Liliumjaponicum* that arrived during 1818. The second painting A/REE/SmV5/114 falls within the batch number 112–117 as HS [Horticultural Society] 143 listed in the The Society’s Drawing Committee’s Minutes as having arrived after 1822 (Charlotte Brooks, pers. comm.).

The Council Minutes recorded on 4 August 1818 included written verification that the lily was, by that time, in the Society’s possession:

“*Mr Sabine stated that he had presented to Sir Joseph Banks in the name of the Society, two bulbs of the*Liliumjaponicum, *recently imported from China by the Society*.”

Whether these bulbs were donated to Banks by Joseph Sabine for Banks’s own Spring Grove House garden in Isleworth or as an additional gift for the Royal Garden at Kew is not known.

Council Minutes for 19 January 1819 relate: “*The Chinese plants which had been entrusted to the care of Mr. William Anderson in the botanic garden Chelsea were ordered to be removed to the Society’s garden and a letter of thanks extended*”.

The Society’s Garden Committee Minutes for 5 March 1819 included: “*Ordered that one pot of*Liliumjaponicum*be presented to each of the nurserymen who are members of the Society*” (Helen Winning, pers. comm.). This statement implies that there were enough bulbs to spare for distribution to the nurserymen from their small rented garden at St. Mary Abbots Place, Kensington. It also confirms that bulbs of the lily will have been in one of Reeves’s two consignments that arrived in 1818, the year before Samuel Brookes’s consignment (see below).

## ﻿Samuel Brookes’s introduction from Canton

Samuel Brookes, a nurseryman of Ball’s Pond Nursery, Newington Green near London, wrote another account of *Liliumjaponicum* in a letter to the Horticultural Society on 2 August 1821, which was published in the fourth volume of the Society’s Transactions (Brookes 1822: 551–553). In this letter, he stated that he and his late partner, Thomas Barr, had imported from China in 1819 a large consignment of the lily that had arrived on board the HEICS “Lady Melville”. The “Lady Melville”, 1263 tons, sailed from London on 16 April 1818 under the command of Captain John Stewart arriving at Whampoa, Canton on 14 September. The return voyage left the Second Bar anchorage, Canton on 25 November 1818 stopping at the south Atlantic island of St Helena for supplies on 3 March 1819 and arrived back in London’s East India Docks on 6 May 1819 https://discovery.nationalarchives.gov.uk/details/r/b23b1f48-af85-4375-8bf6-5c8ae0630ef5. The consignment of bulbs would certainly have been included on board as “Private Trade”. A later report on the difficulties of the transportation of Chinese plants to England written by the EIC surgeon at Canton, John Livingstone, mentioned that Brookes and his partner Barr had actually sent out a collector to Canton in 1819 to locate and bring back plants, although the name of the said collector was not mentioned (Livingstone 1822: 426). This would seem unlikely but may have referred to a possible collaboration with John Reeves.

Brookes reiterated that bulbs of the same lily had been originally sent from China to Kew on board the “Henry Addington” in 1804 and that one plant had flowered at Kew in July 1813, where it was figured by Sydenham Edwards for “Curtis’s Botanical Magazine” as plate 1591. Aiton, however, had described it flowering before 1811 (Aiton 1811: 240). Significantly, Brookes went on to say that all those original plants had since died out, but that bulbs from his own introduction in 1819 and also from another consignment brought in by the Horticultural Society that had also arrived in 1819 were thriving.

Brookes’s mention of the shipments from the Horticultural Society as having arrived in the year 1819 might also be correct as EIC ships may have brought plants including bulbs back that year; however, this was not recorded in the Society’s Minute Book. The next sailing of the “Lowther Castle” did not arrive back from Canton until 9 April 1820, while that of the “Bombay” did not return to Long Reach until 29 September 1820 https://threedecks.org/index.php?display_type=show_ship&id=29088.

## ﻿Chinese Illustrations of *Liliumbrownii*

**Figure 2.**
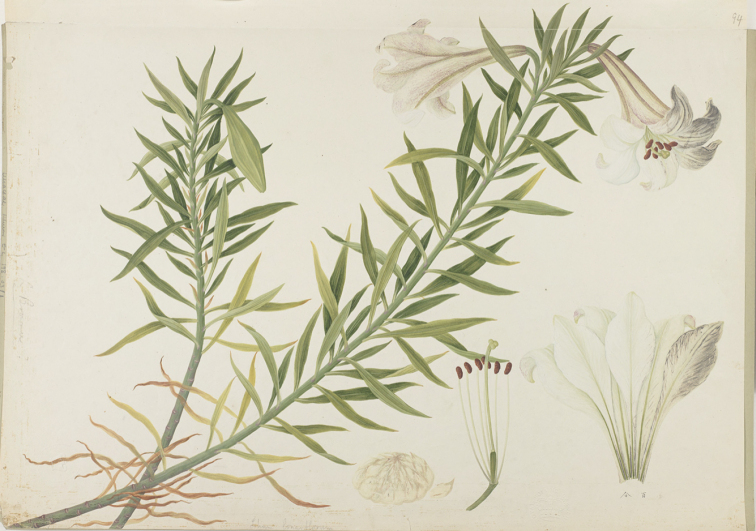
*Liliumbrownii* Illustration number 94 (as *Liliumlongiflorum*) of William Kerr’s drawings for the East India Company conserved at the Royal Botanic Gardens Kew mentioned by Samuel Brookes in Horticultural Transactions vol. 4: 553 (1822).

Samuel Brookes mentioned a drawing of the lily that was in the collection of the East India Company as drawing number 94 (Brookes 1822: 553). It was listed under the Chinese name of “Pa-kup”, a name very similar to the one listed by Kerr in 1804. This has been located in the William Kerr collection of Chinese paintings, now conserved in the archives at the Royal Botanic Gardens Kew. It is catalogued as Kerr Collection *Liliumlongiflorum* number 94 (Fig. 2).

This illustration of *Liliumbrownii* is numbered 94 in the top right hand corner and has two Chinese characters in ink 百合 representing “pa hup” and, in pencil at the bottom, *L.longiflorum* which may have been added later. The illustration is part of two dispatches totalling some 400 Chinese paintings of plants undertaken on behalf of Sir Joseph Banks for the East India Company. One set arrived in 1805 and the second in 1807. There is no indication as to which of these dispatches this illustration belongs. Kerr was tasked by Banks on behalf of the East India Company to find Chinese artists to paint a range of Chinese plants as a putative adjunct to the “Cabinet of Natural Productions” for the India Museum (Jordan Goodman, pers. comm.). This Museum was established in 1801 alongside East India House, the Company headquarters in Leadenhall Street (Goodman and Jarvis 2017: 270, 271). When the India Museum closed in 1879, the natural history drawings were sent uncatalogued to Kew.

The painting must have been undertaken by a Chinese artist under Kerr’s supervision in Macao during its flowering season sometime between June and August. It shows two flowering stems, one with a single bud, the other with two open flowers. There are dissections of the flower showing the six brownish-red stamens, the ovary with the style and stigma attached and the six individual perianth segments. There is also a complete subglobose bulb showing the white scales. Along the margin on one side, “*L.brownii*?” is faintly added in pencil, which must have been added many decades later.

The Asian and African Studies Print Room in the British Library also holds a collection of 309 watercolours of Chinese plants in six volumes that came from the East India Company (NHD52–57). The majority of these were on paper with the Whatman watermark dated 1794. There is no date on any of the watercolours, but there is a sheet of paper amongst the collection with meteorological data on it headed “*Monthly account of the fall of rain at Macao and Canton in China, from September 1807 to July 1809*”. The handwriting on this sheet closely resembles that of William Kerr (Josepha Richard pers. comm.). Two watercolours represent *Liliumbrownii*. The first NDH52/14 has an inflorescence with a single open white flower without showing signs of the reddish colouration on the outside. The lanceolate leaves are bright green and there are individual dissections of the six brown stamens, the ovary with style and stigma attached and the six perianth segments. There is also depicted a squat white bulb and an individual white bulb scale. On the bottom right, in ink in Chinese characters is written “pae hup fa” 百合花 (also written on the reverse in English). In pencil is written “*Liliumjaponicum*” and bottom left in ink “W.Ch”. The origin and purpose of these initials remains a mystery, but might refer to the Chinese name of the artist. The same initials were placed on 152 of the other paintings in the collection.

The second illustration NDH56/25 also has a single inflorescence with one open white flower. This too has the six brown stamens, ovary, style and stigma and six white tepals showing a greenish tinge to the nectaries within. The leaves are shorter and more oblanceolate. There is no name written in pencil in English, but 百合 [“pa hup”] is written in ink in Chinese characters and again in English in pencil on the reverse. At the bottom left, it has the abbreviation “H.Sh.” written in ink. The significance of this is also unknown, but might again refer to the Chinese artist. These initials were placed on 129 of the other paintings in the collection. These two illustrations bear a number of similarities with the Kerr painting at Kew, in particular with respect to the execution of their anatomical dissections. They too must have been undertaken in Macao during the summer months when the plants were in flower.

In his letter on *Liliumjaponicum*, Samuel Brookes stated that a painting of the lily had been prepared by Barbara Cotton in 1820 from the five plants that had flowered from his own consignment and that the painting had been given by him to the Horticultural Society (Brookes 1822: 551). The Society’s Drawing Committee’s Minutes from 1815–1824 included the information that Barbara Cotton (1794–1829), who from 1823 became Mrs Lawrence, had been commissioned to paint a series of paintings of *Lilium* from 1822 onwards. Perhaps, this series of paintings was inspired by the one given to the Society by Brookes? The painting of “*L.japonicum*”, being part of the Miscellaneous Drawings collection, was sold by the Society in 1859 (Charlotte Brooks, pers. comm.).

## ﻿The spread of “*Liliumjaponicum*”

In 1822, the current *L.brownii* was once again mentioned under the name *L.japonicum* as having first arrived in England from China in 1804 by Stephen Reynolds Clarke, although he does not mention from which introduction the description of his plants originated (Clarke 1822: 332). In November 1820, the Horticultural Society sent John Potts from the Society’s Kensington garden to Canton where he met John Reeves and, after a year collecting plants under Reeves’s aegis, he returned in August 1822 having sent back shipments of plants (Elliott 2004: 198). Unfortunately, Potts died shortly after his return, but the Society, undaunted by his death, sent John Damper Parks, this time from the newly-leased garden in Chiswick, out to Canton in April 1823 on the HEICS “Lowther Castle” (Elliott 2004: 200). Parks returned in May 1824 having made contact with John Reeves and having also sent back plants for the Society’s Chiswick garden. Neither the Society’s "Transactions" nor the Council Minute books refer to any lilies having been collected either by John Potts or by John Damper Parks.

The additional introductions of *Liliumbrownii* brought back by Reeves and Brookes, however, soon led to the species becoming widely dispersed. The bulbs crossed the Atlantic to North America where, by 1822, William Prince’s Linnaean Botanic Garden nursery at Flushing, Long Island, New York listed on p. 30: “18. Japan white - *Liliumjaponicum* for $3. 25 cents each.

Meanwhile, in France, according to the French physician and botanist Jean-Louis-Auguste Loiseleur-Deslongchamps, the Chinese species (as *L.japonicum*) was in cultivation in the gardens of Monsieur Cels and Monsieur Boursault and had once again been painted (Loiseleur-Deslongchamps 1822: t. 375). Loiseleur-Deslongchamps mentioned that, although the species had been introduced to England in 1804, it had only recently arrived in France and had flowered for the first time on 10 July 1821. The first of the gardens he mentioned belonged to François Cels (1771–1832), the son of the famous nurseryman Jacques-Philippe-Martin Cels of Petit Montrouge which was then a village just south of Paris. Cels’s garden comprised some 18 acres full of rare plants. The second referred to the garden of the actor, theatre director and revolutionary Jean-François Boursault-Malherbe (1750–1842), whose country house at Yerres, Villeneuve-Saint-Georges, then a small village south-east of Paris, was equally renowned for the rare plants within it and especially for its roses. Both grew the Chinese lily under the name Lis du Japon or *L.japonicum* as depicted in the fine coloured illustration by Pancrace Bessa (Loiseleur-Deslongchamps 1822: t. 375).

A year later, the French botanist Jean Poiret was clearly referring to the Chinese lily under the name *Liliumjaponicum* (Poiret 1823: 21). He stated that the flowers of this lily were larger than others that he had encountered and referred to the exterior of the flower as having a reddish flush. He also reiterated the occasion of its flowering for the first time in 1821 in the gardens of Messieurs Cels and Boursault.

Ten years later, evidence of the success of this lily in cultivation was again illuminated by the beautiful illustration of it as *L.japonicum* by Priscilla Susan Bury (Bury 1831: t. 2). Mrs Bury stated that the plants from which her painting was made had been growing in the Liverpool Botanic Garden for several years. This would have been the garden of the polymath and abolitionist William Roscoe, founded in 1802 near Mount Pleasant, Liverpool, but which is now sadly lost to housing east of Abercromby Square. She also cited Samuel Brookes’ apparent success with the lily by mentioning that his plants had produced three flowers per stem instead of what had previously been reported to be just one. The Liverpool plants she added, were clearly suited to their habitat and were stated to produce five or six flowers per stem. Her mention of Brookes implies that the lilies which he had introduced in 1819 might be the same as those that were still in cultivation in Liverpool by 1830.

## ﻿The naming of *Liliumbrownii*

**Figure 3.**
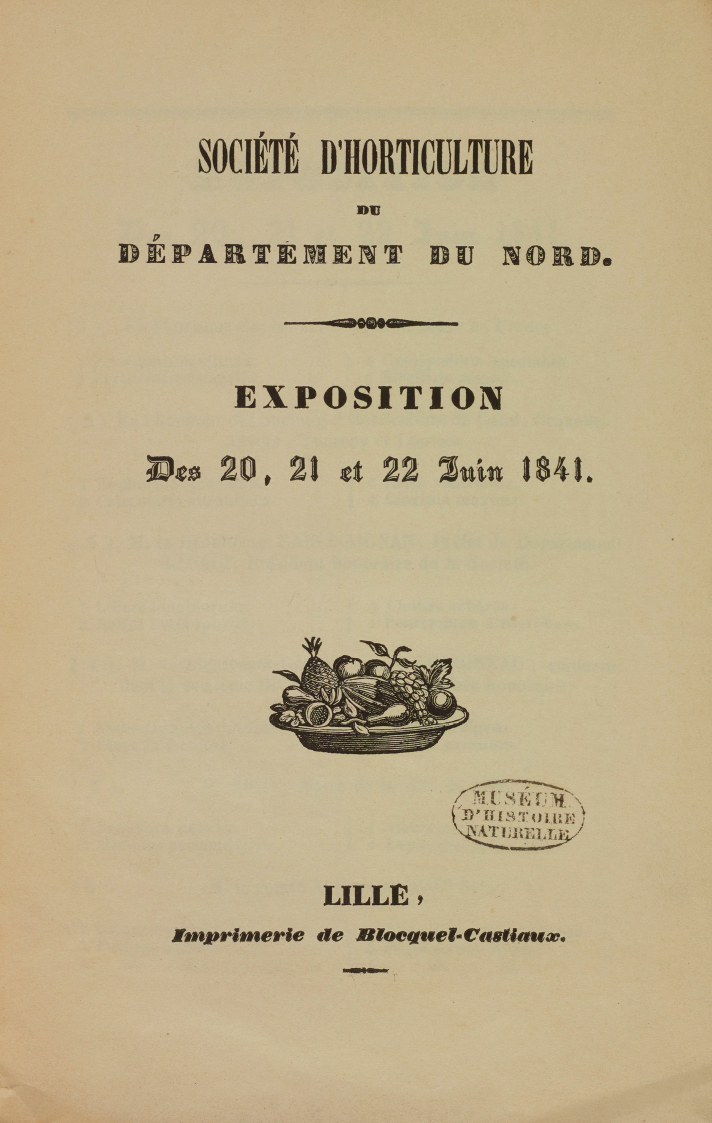
The frequently cited first place of listing of the name *Liliumbrownii* as a nomen nudum in the Société d’Horticulture de Lille (Nord) - 13^th^ Exposition Juin 20, 21 and 22, 1841.

The Chinese lily was finally recognized as distinct from *L.japonicum* and was named *Liliumbrownii* in 1841 (Lemoinier 1841: 7). This was not, however, in the catalogue to an exhibition in Lille, which has been regularly cited to have been its first place of publication (Fig. 3). All reference works which are consistently cited in botanical and horticultural literature refer to the nurseryman F. E. Brown of Slough as being the source of the plant from which the name of the species was derived (e.g. Spae 1845: 138; Spae 1847: 12; Duchartre 1870: 347; Wilson 1925: 28; Woodcock and Stearn 1950: 161; Synge 1980: 161). Also referred to by these writers as the author of the name is the French nurseryman Auguste-Joseph Miellez (1809–1860) of Esquermes-les-Lille, son of Louis Xavier Joseph Miellez (1777–1849), a founder of the Société National d’Horticulture de France in 1825. In neither case, the authority and place of publication is correct. It is correct, however, that the Chinese lily was first included as number 102 in the list of flowers exhibited by the Miellez nursery under the name *Liliumbrownii* in the Société d’Horticulture de Lille (Nord) - 13^th^ Exposition Juin 20, 21 and 22, 1841 (Fig. 3). Miellez’s exhibit, with “(1841)” next to it, signified that 1841 was the first year in which he exhibited that plant, as he had for all other newly exhibited plants. It was entered in the Summer Exhibition in the Bourse [Stock Exchange] in Lille but, as the name lacked any accompanying description in the catalogue, it is a nomen nudum (Art. 38.1 ICN, Turland et al 2018). It was therefore not validly published in that catalogue in spite of the belief by many subsequent writers that it had been (e.g. by Spae 1845: 138; Spae 1847: 12; Duchartre 1870: 347; Wilson 1925: 28; Woodcock and Stearn 1950: 161; Synge 1980: 161). The significance of the date “1841” in this exhibition catalogue means that the reference by the writers cited above to Miellez’s catalogue will have been to the catalogue of Miellez’s plants which were exhibited for the first time at the exhibition in Lille, not to the publication of the name in any nursery catalogue produced by Miellez. Moreover, there do not appear to be any extant Miellez nursery catalogues of that period despite a comprehensive search for them.

Six years later, Charles Morren, editor of “Annales de la Société royale d’agriculture et de botanique de Gand”, reviewed the work of Dieudonné Spae praising his colleague (Spae 1845: 438, t. 41) for his full and accurate description of *Liliumbrownii* (Morren 1847: 309). Morren went on to include a highly critical note concerning the taking up of names published without any adequate description and in an unscientific manner. Morren made this point entirely with reference to the inadequate naming of plants in catalogues, such as in the one for the Lille Summer Exhibition in which *Liliumbrownii* was merely listed (Morren 1847: 309). According to current rules of the ICN, however, Spae (1845) was beaten to it by an earlier description (Art. 11.3).

**Figure 4.**
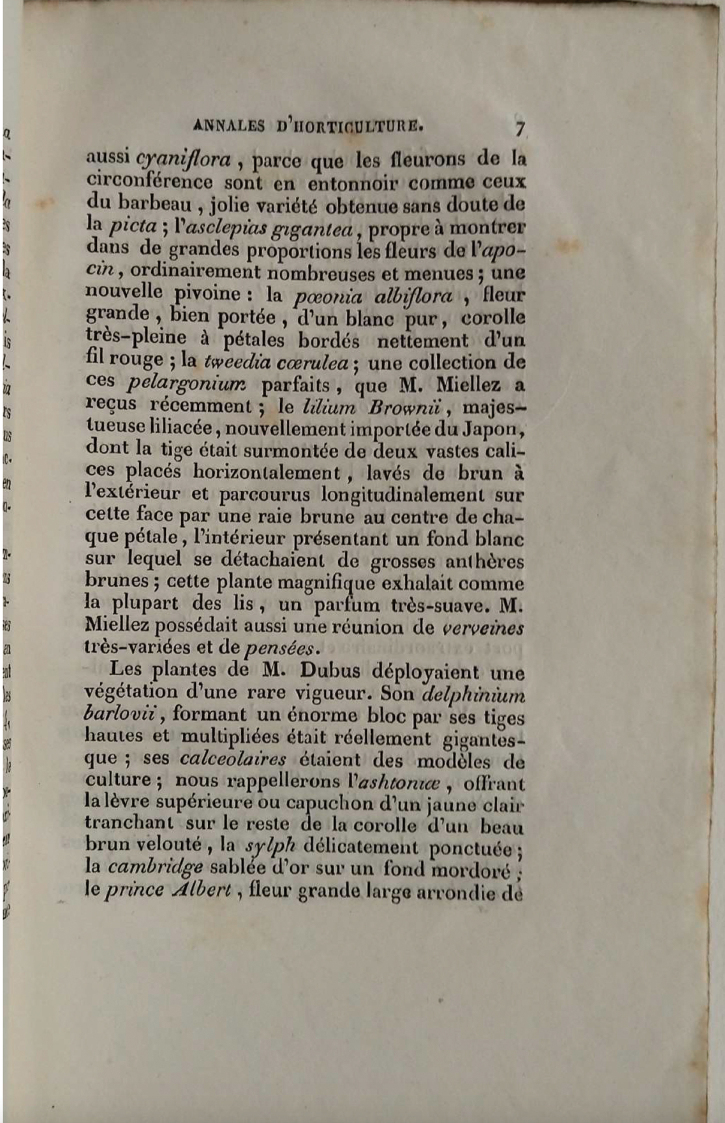
The first validly published description of *Liliumbrownii* A.Lemoinier in Annales de la Société d’Horticulture du Département du Nord (Lille) 13: 7 (1841).

The first valid description of *L.brownii* was published four years before Spae’s and was in the report of the Summer Exhibition in Lille in the first cahier (issue number 1) of the “Annales de la Société d’Horticulture du Département du Nord (Lille)” 13: 7 (1841) (Fig. 4). The description (translated from the French) is:

“*Mr. Miellez has received recently the*" Liliumbrownii, *majestic liliaceae, newly imported from Japan, whose stem was surmounted by two vast calyces placed horizontally, washed with brown on the outside and traversed longitudinally on this face by a brown stripe in the centre of each petal, the interior with a white background from which protrude large brown anthers; this magnificent plant exhaled, like most lilies, a very sweet scent.*”

That report, however, was unsigned, but according to the “Annuaire statistique du Département du Nord 14th Année” -1842 (Demeunynck and Devaux 1842: 379), the secrétaire-adjoint, who would also have been the editor [rédacteur] of the “Annales de la Société d’horticulture du Département du Nord” in 1841, was Auguste Lemoinier. The correct authorship and place of publication of this name is, therefore, *Liliumbrownii* A.Lemoinier, Ann. Soc. Hort. Dép. N. 13: 7 (1841). Lemoinier was cited as secrétaire-adjoint for the following year in the “Annuaire statistique du Département du Nord 14^th^ Année” -1842 (Lemoinier 1842: 379).

## ﻿The name *Liliumbrownii* becomes established

As the appeal of *Liliumbrownii* spread across the Continent of Europe, it was inevitable that celebrated writers on all matters horticultural proceeded to describe and/or illustrate this highly ornamental species (e.g. Poiteau 1844: 496; Spae 1845: 438, tab. 41; Lemaire 1845: 257 + tab.; Van Houtte 1845: 22; Spae 1847: 12; Lemaire 1848: 74 + tab.; Duchartre 1870: 342).

Shortly after its first appearance under the unpublished name *L.brownii* by Miellez on the exhibition table in Lille, the lily was exhibited two years later as *L.brownii*. There was no description and it was listed as number 2569 by the nurseryman Jean van Geert of Gand [Ghent], in Belgium. Van Geert exhibited it in the Catalogue de l’Exposition de la Société Royale d’Agriculture et de botanique de Gand (Anon 1843: 42). In France, the botanist Pierre-Antoine Poiteau also recognised that a name change for the lily was required from the continued use of *L.japonicum* to *L.brownii*, publishing this proposal in the fifth volume of the influential Revue Horticole (Poiteau 1843: 406). The following year, the liliophile Belgian botanist Dieudonné Spae exhibited the lily under the name *L.brownii* with “(*L.japonicum*)” as its synonym, again without description, under exhibit number 1244 in the 76^th^ Exposition de la Société Royale d’Agriculture et de Botanique de Gand in March 1844 (Anon 1844: 25). Three years later, another mention of the lily as *L.brownii* was by Pierre Denis Pépin who stated that the nurseryman Louis Thibault had sent to the Société d’horticulture a superb plant of this species with its white tubular flowers washed with purple on the outside (Pépin 1847: 345). Louis Thibault had only just formed a partnership with Jean-Baptiste Keteleer at Sceaux near Paris in order to grow many rare and exotic plants. The lily was by then, well established in cultivation and at long last was becoming recognised under the correct name.

*Liliumbrownii* was eventually included by Henry John Elwes in his superb Monograph on the genus *Lilium*, accompanied by a beautiful illustration by Walter Hood Fitch (Elwes 1877: t. 8).

## ﻿The Brown nursery of slough

Few accurate records exist of the Nursery known as Browns of Slough in the 18^th^ and early 19^th^ centuries because all documents relating to them were destroyed in a catastrophic fire in Thomas Brown’s house in 1840 (Dean 1885: 264). Census records, births, marriages and deaths in the National and Parish registers, wills in the National Archives and numerous articles in the horticultural and local press have helped to fill in some of the missing data presented here.

Thomas Brown (1748–1814) founded a nursery at Upton-cum Chalvey in 1774 on the fertile and well-drained soil of the Thames Valley alongside the Great West Road from London to Bristol (Fraser Maxwell 1973: 100). The nursery was just to the east of the small village of Slough, then in the county of Buckinghamshire and was a major exhibitor of plants to the Salt Hill Floral Society established in Slough in 1783. His son Thomas Harper Brown (1777–1817) married Elizabeth Penny (1780–1833) and, together, Thomas father and son and the son’s wife Elizabeth ran the nursery. Thomas Harper Brown and Elizabeth had several children of whom the oldest were Thomas (b. 1804), Edward, (b. 1805) and John (b. 1807).

Thomas Harper Brown died in 1817. According to the terms of his will, the nursery was to be left in the hands of his cousin Charles Brown (1796–1836) of Alpha Cottage, Slough in a partnership with Thomas’s widow Elizabeth. The partnership between Charles and Elizabeth was to remain in place until Elizabeth's sons reached the age of 21. In 1833, Elizabeth Brown died leaving the nursery in the hands of Charles Brown who was joined in 1834 by his young cousin Thomas upon his reaching 21 and the following year by Thomas’s younger brother Edward (the youngest brother John having died in 1824). In 1836, Charles Brown died aged just 40 leaving the brothers Thomas and Edward as partners in the nursery business of Messrs Brown of Slough.

Charles Brown became a leading light in the nursery world, specialising in breeding and exhibiting dahlias, roses, heartsease and tulips. He was elected a member of the prestigious Horticultural Society of London on 6 July 1819 (Helen Winning pers. comm.). In 1833, Charles Brown was awarded two Banksian medals from the Horticultural Society for his exhibits of heartsease and tulips (Bentham 1835: 534). Charles and his cousin’s wife Elizabeth also exhibited as E & C Brown of Slough at the local Salt Hill Society’s flower show and, encouraged by the florist George Glenny, also exhibited for the Metropolitan Society of Florists and Amateurs whose Patroness was Queen Adelaide. The latter was founded in 1832 as a rival to the Horticultural Society of London (Elliott 2001: 172). Charles Brown married Sarah Botham and the Brown nursery floral exhibits were regularly included in the Metropolitan Society shows held in the garden of Botham’s Hotel, Salt Hill, Slough. This Hotel was one of the grandest along the fashionable Great West Road from London to Bath (Glenny 1834: 28; Fraser Maxwell 1973: 72).

After Charles’s death in 1836 (Anon 1836: 5), Thomas and Edward Brown maintained a partnership at Slough and Salt Hill as Messrs Brown of Slough. The brothers also maintained a seed shop in the Egyptian Hall, Piccadilly, London until 1841 (Brown and Brown 1841: 762). In March 1842, Edward Brown announced that he was leaving the business and dissolving the partnership. There was a subsequent sale of the stock within the 14 acres of the Hencroft Nursery which belonged to Edward (Anon 1842: 138). Thomas Brown was, thenceforth, the sole owner (Brown 1842: 601) and was still listed under the category of nursery and seedsmen in Pigot’s Directory (Slater 1844: 50). Edward Brown, meanwhile, seems to have capitalised on his property assets. No doubt as a result of his nursery credentials, he was chosen to be the secretary of a Testimonial fund in 1864 to Mr. Thomas Ingram, head gardener at the Royal Gardens, Frogmore (Brown 1864: 1129).

Thomas Brown was elected a Fellow of the Horticultural Society on 20 September 1836 (Helen Winning pers. comm.) and, less than a month later, he married Mary Ann Rhodes on 15 October 1836. Thomas Brown exhibited plants at the Horticultural Society’s shows from 1838 to 1844 (e.g. Loudon 1838: 543–544; Brown 1843: 641; Anon 1844: 375). There is, however, an advert signalling the end of Thomas Brown’s tenure at Slough due to his failing health which stated that he was submitting for sale his magnificent tulip collection at the Slough Nursery through his agent Mr George Glenny at the Gardener’s Gazette Office (Glenny 1845: 282). He sold the contents of his house and advertised the contents of his nursery for sale in July 1845. It seems that Thomas Brown then leased the nursery to a partnership of William George Cutter and George Shanklie in 1845 which was then dissolved in 1848. Thomas Brown then sold the nursery which was, by then, known as the Royal Nursery Slough to his former foreman Charles Turner (1816–1885), in December 1848. Thomas emigrated with his wife Mary Ann and his three sons and a daughter to Hawaii where he served as Recorder of Deeds and died in Honolulu in 1886 (Robinson 1886: 598). Turner maintained the nursery into the mid- and later 19^th^ century (Anon 1885: 12).

There clearly was never any F. E. Brown who was associated with this nursery and any attribution to a Mr F. E. Brown of Slough in relation to *Liliumbrownii* is an error. It is probable that the original source of this error was Dieudonné Spae who wrote “*il a fleuri pour la première fois chez MM. F. E. Brown, à Slough près de Windsor*” (Spae 1845: 138). It is also clear from the many attributions to “Messrs Brown of Slough, Charles Brown of Slough and Thomas and Edward Brown of Slough” that these were, without doubt, the nurserymen for which the species was named. How then did *Liliumbrownii* arrive at this nursery? Charles Brown was a fellow judge with Donald Munro, the head of the ornamental section of the Horticultural Society of London’s Chiswick garden when floral exhibits were held at their horticultural shows (Anon 1832: 4). There is a coincidence that occurred at a show in the Horticultural Society’s Hall in Regents Street, London on 2 July 1833. At that show, Charles Brown exhibited a large bunch of one of his roses ‘Brown’s Superb’ and a huge collection of 120 of his heartsease (*Viola* hybrids), while, in the same show, *Liliumjaponicum* was also exhibited by Donald Munro on behalf of his employer, the Horticultural Society (Loudon 1833: 508). As there was no description of the exhibit, one can only assume that this was the Chinese lily and not the Japanese *L.japonicum*. It is not beyond the realms of possibility that Charles Brown could have acquired the lily from his colleague. *Liliumjaponicum* was again exhibited the following year on 5 July 1834 in the Society’s Chiswick garden (Paxton 1834: 381).

Alternatively, perhaps it may have been acquired during the first year of the partnership of brothers Thomas and Edward Brown, following the death of Charles Brown in 1836? Thomas had been elected a Fellow of the Horticultural Society in 1836, so when *Liliumjaponicum* was once again exhibited by Donald Munro for the Horticultural Society on 18 July 1837, did Thomas acquire the lily then (Loudon 1837: 478)? Thomas Brown was certainly known to have exhibited several species of *Lilium* at the Chiswick Horticultural Show on 4 July 1840, although neither the name *L.japonicum* nor any other lilies names were specifically mentioned (Marnock 1841: 60).

## ﻿The French connection – Pépinières Miellez

It was widely reported that *Liliumbrownii* was introduced to England circa 1835 or 1836, where it was acquired by Messrs Brown of Slough near Windsor (Spae 1845: 438; Spae 1847: 12; Duchartre 1870: 342; Van Eeden 1876: t. 63). This acquisition by Messrs Brown is unlikely to have been from an unverified later introduction of the species from Canton. It has been suggested that it was Thomas Brown who was responsible for the importation of the bulbs directly from China (Fraser Maxwell 1973: 99), but this is highly unlikely due to the stringent regulations imposed on foreign regimes by the Chinese at that time as have already been explained above.

In the Horticultural Society of London’s Council Minutes, dated 2 July 1830, there is the statement: “*Ordered that Mr. Reeves be written to, to discontinue the importations and drawings now forwarded by him to the Society*”. By 1831, the Society was in great financial difficulties and keen to save money in whichever way possible. One small way for them to do this was to stop the expense on the importation of plants and drawings. John Reeves left Canton to finally return to England in 1831, which coincidentally was only two years before the EIC lost its monopoly in China through the Charter Act 1833. John Reeves had been joined in Canton in 1824 by his son John Russell Reeves (1804–1877), who remained as the last EIC Tea Inspector in Canton until 1838 and was known to have sent some plants back to England. There is no evidence to suggest that *Liliumbrownii* was amongst them, but that possibility cannot be ruled out.

According to the reports mentioned by Spae (1845) and van Eeden (1876), three bulbs of the lily were acquired in 1837 from Brown of Slough by Monsieur Auguste Miellez at Esquermes for his nursery which was, at that time, in a district just to the southwest of Lille in northeast France. Reports suggested that M. Miellez had imported them into Belgium the following year [1838?] under the name *L.brownii*, so named by him in honour of those who first flowered the species (Van Eeden 1876: t. 63). Thence, it seems the lily was communicated to Herman Shuurmans-Stekhoven (1757–1839), the head gardener of the Leiden Botanic Garden in The Netherlands (Spae 1847: 12).

The question then arises as to how Auguste Joseph Miellez pépinière [nurseryman] of Lille actually acquired the bulbs and how there may have been a link with the Slough nursery? Owing to the absence of reliable records following the disastrous fire in Thomas Brown’s house in 1840, any suggestions as to how the lily bulbs might have crossed the Channel must be pure speculation. The nursery of Louis Xavier Joseph Miellez and his son Auguste Miellez was famous for the breeding and cultivation of roses. Charles Brown of Slough was also a well-respected breeder of roses as mentioned by John Claudius Loudon (Loudon 1831: 66) and by the rosarian and nurseryman Thomas Rivers of Sawbridgeworth, Hertfordshire (Rivers 1838: 17). Thomas Brown followed in his cousin Charles’s footsteps after Charles’s death in 1836, specialising in the breeding and exhibition of dahlias, but he also bred roses, heartsease, tulips and pinks.

It is possible that Auguste Miellez had heard of the lily via his nursery colleagues and had simply asked for them to be sent to him or, alternatively, he may have made a visit across the Channel on a 400 mile (ca, 640 km) return journey to Slough in search of new plants for his nursery. It is also possible that Charles Brown may, perhaps, have gone the other way offering the three bulbs and one of his roses in exchange for one of Miellez’s fabulous French roses. That journey either way may have also taken place after Charles Brown’s death in 1836 and during the tenure of the brothers (“les frères T & E”) Thomas and Edward Brown. Any further evidence, if it still exists and comes to light, may fill in this small piece of the puzzle.

## ﻿*Liliumodorum* Planch.

A further complication arose with the history and description of the Canton lily under yet another species name *Liliumodorum* Planch. (Planchon 1854: 53 t. 876). The plant described by the French botanist Jules Emile Planchon under the name *L.odorum* has lanceolate leaves and white flowers stained with deep red externally and with deep red along the mid-ribs. There is no doubt this is *L.brownii*.

Planchon stated that two different species were known under the name *L.japonicum*, one was *L.japonicum* of Thunberg and the other was *L.brownii* Hort. which, at first sight according to Planchon, was very similar to *L.odorum* (Planchon 1854: 53). He had examined a sheet of *L.japonicum* collected by Thunberg in Japan and which was conserved in the [Jules Paul] Benjamin Delessert Herbarium in Geneva. He noticed that the leaves on the Thunberg specimen were distinctly petiolate and concluded that the plant introduced by Captain Kirkpatrick of the East India Company and which was subsequently described by various authors under the name *L.japonicum* was not Thunberg’s plant. He believed the latter, which was also figured in Loddiges Botanical Cabinet with a plate as *L.japonicum* (Loddiges 1820: t. 438), was identical with his *L.odorum*. Having made that statement, he added a short footnote “*Cette plante serait-elle le*Liliumbrownii? *Mais les antheres plus courtes semblent la rapprocher davantage de notre*L.odorum.” [could this plant be *Liliumbrownii*? The shorter anthers seem to bring it closer to our *L.odorum*]. Planchon added that *L.odorum* can be distinguished [from *L.brownii*] by the narrower leaves, less strongly scented flowers and the longer anthers (Planchon 1854: 53). The accompanying illustration of *L.odorum* by Louis Stroobant, painted from a specimen growing in Louis van Houtte’s nursery, also includes as a synonym *L.japonicum* Lodd. (non Thunb.). The morphological distinctions described by Planchon, however, all fall within the range of *L.brownii* and no mention is made of the origin of the plant he described and had figured, although “Japan - châssis froid” [cold frame] is written on the illustration.

Eduard Regel in Zurich very quickly picked up on Planchon’s new species name. In July that year, under the heading Neue Zierpflanzen [new ornamental plants], he stated that *L.japonicum* with its petiolate leaves is unlikely to still be in cultivation. He reiterated Planchon’s point that *L.odorum* is the plant depicted in Loddiges Botanical Cabinet under the name *L.japonicum* (Loddiges 1820: t. 438) and that *L.brownii* is closely related, but the flower is comparatively odourless (Regel 1854: 234–235).

## ﻿Taxa related to *Liliumbrownii*

Taxonomists in the past have found difficulty in diagnosing the morphological differences between those species of *Lilium* with infundibiliform or funnel-shaped flowers (Duchartre 1870; Baker 1875; Elwes 1877; Franchet 1892; Cavalerie 1911). These scholars used such characters as leaf shape and length, perianth shape and colouring and glabrous or pubescent nectaries, filaments and style bases in order to delimit the taxa and found them to be variable and, therefore, inconsistent.

Several molecular DNA-based studies using both plastid and nuclear markers have helped resolve some of the relationships amongst these species (Nishikawa et al. 2001; Lee et al. 2011; Du et al. 2014; Gao et al. 2015; Huang et al. 2018; Givnish et al. 2020). These molecular studies have shown that the Asian species of *Lilium* with trumpet-shaped flowers belong in two clades: One comprises *Liliumbrownii*, *L.formosanum* A.Wallace, *L.longiflorum* Thunb., *L.neilgherrense* Wight, *L.philippinense* Baker and *L.wallichianum* Schult. & Schult.f. These species all have bulbs with either white, ivory or yellow coloured bulb scales, which, on exposure to air, exhibit a pinkish or light brownish colour. In addition, the inner basal section of the corolla in all these species is greenish-white or ivory-white, not yellow. Two more recently described Chinese species also share many of the same characters as *L.brownii* with white or pale yellow bulb scales. These are *L.anhuiense* D.C.Zhang & J.Z.Shao, Acta Phytotax. Sin. 29(5): 475 (1991) and *L.wenshanense* L.J.Peng & F.X.Li, Acta Bot. Yunnan., Suppl. 3: 33 (1990). *Liliumanhuiense* was distinguished from *L.brownii* by the foliar bracts at the apex of the inflorescence axis being curved as opposed to straight and by the style bases being pubescent as opposed to glabrous. These are, however, variable characters across the range of the species. *Liliumwenshanense* was distinguished from *L.brownii* by the bulbs having segmented rather than entire scales. This character too has been found to be inconsistent (Gao and Gao 2014: 102). These two species have also been shown to belong on the same clade as the other species with predominantly white or yellow bulb scales (Huang et al. 2018) and are, therefore, placed here into the synonymy of *L.brownii*.

The second clade comprises those species with pink to dark reddish-purple, sometimes almost blackish bulb scales when fresh and have corollas that are richly yellow within. These have been placed in Liliumsect.Regalia Baranova, *Novosti Sist. Vyssh. Rast*. 8: 94 (1971): *L.leucanthum* (Baker) Baker, *L.sulphureum* Baker ex Hook.f., *L.sargentiae* E.H.Wilson, *L.regale* E.H.Wilson and *L.centifolium* Stapf.

## ﻿Typification of *Liliumbrownii*

At no stage in its botanical history has a type been allocated to the species. The liliophile Kew botanist John Gilbert Baker segregated L.browniivar.viridulum from (by implication) var. brownii on the shorter, wider, more oblanceolate leaves and paler greenish colouration on the outside of the corollas with less pronounced claret markings (Baker 1885: 131). Baker’s statement “The leaves are much broader and shorter than in the type” is almost certainly intended to refer to what he regarded as the typical variety of *L.brownii*. This point is strengthened by his citation of (Mielle) [i.e. Miellez] as the author of the name and was accompanied by a reference to the description and illustration in *Flore des Serres* by Charles Lemaire. The latter portrays a plant with linear-lanceolate leaves and a flower with reddish markings on the outside of the perianth (Lemaire 1845: t. 47). These references, however, do not constitute typification of the species. William Stearn regarded what he called L.browniivar.brownii as being based on L.japonicumvar.brownii (Spae) Baker (Baker 1871: 709); however, he again did not indicate any type specimen or illustration (Stearn 1948: 5).

The neotype chosen here for the name *Liliumbrownii* is a collection by Pierre Julien Cavalerie from Guizhou Province, China https://data.rbge.org.uk/search/herbarium/?specimen_num=956330&cfg=zoom.cfg&filename=E00934044.zip. Cavalerie was clearly confused as he described L.browniivar.brownii as a variety of *L.longiflorum*. He compared it to what he had already just referred to as *L.brownii*, but which, according to his description, “la tige bulbifere chez les jeunes sujets qui n’ont pas des fleurs” was in fact *Liliumsulphureum* Baker ex Hook.f.. His description of what he referred to as *Liliumlongiflorum* but was described by Lévéille as L.longiflorumvar.purpureoviolaceum (i.e. L.browniivar.brownii) included the statement “La fleur un peu plus petite, plus ouverte, à divisions plus minces est intérieurement blanche et extérieurement d’un violet très variable bien que le blanc domine. Ce lis fleuret deux mois plus tôt que le *L.brownii*; il est commun au sud de Pin-Fa” [The somewhat smaller, more open flowers with narrower divisions is white internally and with very variable purple markings outside on a white background. This lily flowers two months earlier than *L.brownii*; it is common near Pin-Fa] (Cavalerie 1911: 245).

The neotype is based on one of two Cavalerie collections at E from this locality described by his friend Augustin Abel Hector Léveillé under the name Liliumlongiflorumvar.purpureoviolaceum H.Lév. in 1909. This sheet fits well with the protologue of the name. Lemoinier’s mention of the large white flowers washed with brown externally and with a dark brown stripe along the mid-rib equate to the dark purplish colouring of the variety published by Hector Léveillé (Léveillé 1909: 264). In fact, the colouring lies somewhere between brown and purple. The lanceolate leaves which barely shorten up the inflorescence axis clearly refer to L.browniivar.brownii and not to the oblanceolate leaves that quickly shorten to obovate as they extend up the axis in L.browniivar.viridulum Baker.

There is a useful representative illustration of L.browniivar.brownii (Fig. 1) in Curtis’s Botanical Magazine (Ker-Gawler 1813: t. 1591). The plant in the illustration exhibits leaves which are lanceolate and remain more or less consistent in length up the inflorescence axis and the corollas have a pronounced dark purplish flush on the three outer perianth segments. Liliumbrowniivar.chloraster (Baker) Baker has greenish corollas and lanceolate leaves, whereas L.browniivar.viridulum Baker, although having a distinct brownish-red flush on the outside of the corolla, has oblong-lanceolate leaves that decrease markedly to obovate in length up the inflorescence axis.

**Note**: The article in Gardeners’ Chronicle, in which the names *Liliumaduncum*, *L.austral*e, L.browniivar.ferum and L.browniivar.primarium were first published, was written by Elwes (Elwes 1921: 100–101). However, the key to the taxa, associated with *L.brownii* as well as the five additional adnotations, were quoted directly from a letter written to Elwes by Otto Stapf in Kew. According to Elwes, this letter was sent to him following his request for clarification on the status of one of Kew’s specimens. The key and adnotes by Stapf are clearly indicated by the enclosing quotation marks within Elwes’s paper. Stapf is, therefore, the author of these names within this article. As there are no specimens at K annotated by Stapf attributed to the name Liliumbrowniivar.brownii and he did not include that varietal name in his key, L.browniivar.primarium Stapf is considered to refer to typical L.browniivar.brownii. Moreover, with respect to a choice of type material, Stapf did not include the term “typus” or its equivalent (Art. 7.11). Gao and Gao (2014: 102) attempted to neotypify *L.brownii* on a Cipriano Silvestri specimen from Hubei *Silvestri 199*, July 1904 conserved in FI; however, they did not cite “designated here” or “hic designatus” and, therefore, being published after 2000, this putative typification also does not satisfy the requirements of Art. 7.11, hence the need for the neotype designation in this paper.

Further invalid names or later homonyms are:

- *Liliumbrownii* Miellez, Cat. Exposition 20–22 Juin Société d’horticulture de Lille: 9. (1841) nom. nud.

- *Liliumbrownii* Poit. & A.Vilm., Rev. Hort. Ser. 2(2) vol. 5: 495 (1844)

- *Liliumbrownii* Spae, Ann. Soc. Roy. Agric. Gand 1: 437 (1845)

- Liliumjaponicumvar.brownii Siebold, Catalogue 1870–1871: 51 nom. nud.

- Liliumjaponicumvar.colchesteri Van Houtte, Fl. Serres Jard. Eur. 21: 73 (1875) nom. nud.

### ﻿ Lilium brownii

Taxon classificationPlantaeLilialesLiliaceae

A.Lemoinier, Ann. Soc. Hort. Dép. N. 13: 7 (1841).

43620CB4-0E09-5364-9C4C-037F1792ED8C

#### Neotype designated here.

China, Guizhou Province, Pin-fa, 26 June 1907, *P.J.Cavalerie* s.n. (neo. E!) [E-00934044]. Note: this is also the holotype of Liliumlongiflorumvar.purpureoviolaceum H.Lév. See also above under typification. https://data.rbge.org.uk/search/herbarium/?specimen_num=956330&cfg=zoom.cfg&filename=E00934044.zip

### ﻿Key to the varieties of *Liliumbrownii*

**Table:** 

1	Leaves linear to lanceolate, reducing only slightly in length towards the apex of the inflorescence axis	**2**
–	Leaves oblanceolate to obovate, reducing markedly in length towards the apex of the inflorescence axis; corollas tinged externally with only a faint dash of claret-brown on outer tepals	**Liliumbrowniivar.viridulum**
2	Corollas ivory white tinged externally with claret-brown with a pronounced dark streak along the mid-ribs of each outer tepal	**Liliumbrowniivar.brownii**
–	Corollas ivory white tinged greenish externally especially along tepal mid-ribs	**Liliumbrowniivar.chloraster**

### ﻿ Lilium brownii var. brownii

Taxon classificationPlantaeLilialesLiliaceae

1215230B-0E42-58B0-BE44-422FF8461B86

≡ Liliumjaponicumvar.brownii (A.Lemoinier) Baker (as L.japonicumvar.brounii), Gard. Chron. 1871(1): 709 (1871).
≡ Liliumbrowniivar.primarium Stapf in Elwes, Gard. Chron., ser. 3, 70: 101 (1921) – See Note above under typification.
= Liliumodorum Planch., Fl. Serres Jard. Eur. 9: 53 (1853–1854) Lectotype designated here [Icon]: t. 876 Fl. Serres Jard. Eur. 9 (1853–1854)
≡ Liliumbrowniivar.odorum (Planch.) W.Watson, The Garden 47: 97, (1895).
= Liliumlongiflorumvar.purpureoviolaceum H.Lév., Repert. Spec. Nov. Regni Veg. 6: 264 (1909). Holotype: China, Guizhou, Pin-fa, 26 June 1907, *P.J.Cavalerie* s.n. (holo. E!) [E-00934044]; paratype: China, Guizhou, Pin-fa, 13 Feb 1902, *P.J.Cavalerie* 448, (para. K!).
= Liliumaustrale Stapf in Elwes, Gard. Chron., ser. 3, 70: 101 (1921). Lectotype designated here from syntypes: China, Hong Kong, (as Liliumlongiflorum) 1847, *Captain Champion* 23 (lecto. K!) [K-000464652]; isolectotype: China, Hong Kong, (as Liliumlongiflorum) sheet labelled 23 (isolecto. K!) [K-000464653]; isolectotype: China, Hong Kong (as Liliumlongiflorum) without collector, but with number “23”, without locality or date (isolecto. K!) [K-000464655] !); syntypes: China, Hong Kong, (as Liliumlongiflorum) top of ridge, 28 June 1859 “Colonel Urquhart”, sheet labelled 200 (syn. K!) [K-000464654
≡ Liliumbrowniivar.australe (Stapf) Stearn, Lilies of the World: 165 (1950).
= Liliumbrowniivar.colchesteri E.H.Wilson, Lilies East Asia: 30 (1925). Lectotype designated here: [Icon] Bot. Mag. 38: t.1591 (1813) as L.japonicum non Thunb.
= Liliumanhuiense D.C.Zhang & J.Z.Shao, Acta Phytotax. Sin. 29: 475 (1991). Holotype: China, Anhui, Shitai, Guniujiang, 1800 m alt. 18 June 1983, *Shao Jian-Zhang* 8350111 (ANUB).

#### Description.

A variable species with a wide distribution across central and southern China. Three varieties are recognisable.

*Bulb* subglobose frequently slightly flattened 2–5 × 2–7 cm, scales white, ovate, thick, sometimes articulated; *stem* 70–200 cm, green or reddish tinged, smooth or papillose, rooting at base when growing; *leaves* scattered, sessile, linear, lanceolate, (oblanceolate or obovate-lanceolate in var. viridulum) (5) – 16 × (0.6) – 2 cm, glabrous, dark green, paler beneath, 3–7 veined, margins entire or undulate; *inflorescence* 1–7 flowered, subumbellate; *pedicels* 3–6 cm long, glabrous; *flowers* horizontal, slightly to strongly fragrant, tepals spreading gradually from the base, recurved at apex, ivory white within, externally suffused or finely speckled with reddish-purple, especially on the three outer tepals, often with pronounced reddish-purple colour along mid-ribs (greenish externally in var. *chloraster*) 13–18 × 2–4 cm; inner tepals 13–18 × 3.5–5 cm; nectaries linear, green, papillose or subglabrous along margins; *stamens* 10–13 cm long, slightly upwardly curving, glabrous or papillose at base, anthers versatile, linear, brown or orange-brown, pollen cinnabar to reddish-brown; *style* 9–11 cm long, glabrous or pubescent at base, stigma 6–8 mm across, trilobed, pale greenish-yellow; *capsule* 4–6 × 3–4 cm, cylindrical, six-ribbed.

#### Distribution.

**China**: Anhui, Fujian, Gansu, Guangdong, Guangxi, Guizhou, Henan, Hubei, Hunan, Jiangxi, Shaanxi, Sichuan, Yunnan, Zhejiang.

#### Ecology.

Growing in open grassy meadows, rocky hillsides, open woods and amongst low scrub, 100 to 2200 m alt. Flowering in June to August.

#### Illustration.

http://apps.kew.org/herbcat/getImage.do?imageBarcode=K000464654 (as *Liliumaustrale*)

### ﻿ Lilium brownii var. chloraster

Taxon classificationPlantaeLilialesLiliaceae

(Baker) Baker, Gardeners Chronicle ser. 3 vol. 10: 225 (22 August 1891)

26D1FDBD-B636-5C99-9A34-66DD9DE67E35

≡ Liliumbrowniivar.chloraster (Baker) Baker, Gardeners Chronicle ser. 3 vol. 10: 225 (22 August 1891)
≡ Liliumchloraster (Baker) E.H.Wilson, Journal of the Royal Horticultural Society vol. 42: 36 (1916)
≡ Liliumleucanthumvar.chloraster (Baker) E.H.Wilson, Lilies East Asia: 41 (1925).
= Liliumwenshanense L.J.Peng & F.X.Li, Acta Bot. Yunnan., Suppl. 3: 33 (1990). Holotype: China, Yunnan, Wenshan “in pratis 1000–2200 m” (Cultivated Kunming Botanic Garden), 30 June 1989, *L.J.Peng* 89-1 (holo. KUN!), KUN304310 [barcode KUN-1219367]; isotype: China, Yunnan, Wenshan (Cult.) (iso. KUN!), KUN304309 [barcode KUN-1219364].

#### Basionym.

Liliumlongiflorumvar.chloraster Baker, Gardeners Chronicle ser. 3 vol. 10: 66 (18 July 1891) **Holotype**: China, Hubei, *A.Henry* s.n. (Cult. July 1891, RBG Kew, floral parts in two capsules) via Charles Ford in Hong Kong (holo. K!) [K-000464716]

#### Diagnosis.

Differing from var. brownii by the greenish colouration on the outside of the corolla. *Liliumwenshanense* was differentiated by having articulated scales, a feature now found to vary across the range of the species.

#### Distribution.

**China**: Anhui, Fujian, Gansu, Guangdong, Guangxi, Guizhou, Henan, Hubei, Hunan, Jiangxi, Shaanxi, Sichuan, Yunnan, Zhejiang.

#### Ecology.

Growing in open grassy meadows, rocky hillsides, open woods and amongst low scrub, 100 to 2200 m alt. Flowering in June to August.

### ﻿ Lilium brownii var. viridulum

Taxon classificationPlantaeLilialesLiliaceae

Baker, Gard. Chron. 24: 134 (1 August 1885)

64D7B725-45C3-5DC7-B4B9-86AF6E67A86D

= Liliumbrownii [unranked] *brevifolium* T.S.Ware ex Rob., The Garden 28: 115 (1 August 1885). Type not found.
= Liliumbrowniivar.platyphyllum Baker, Gard. Chron. ser. 3, 10: 225 (1891). Type not found: China, Hubei, *A.Henry* s.n.
= Liliumaduncum Stapf in Elwes, Gard. Chron., ser. 3, 70: 101 (1921). Lectotype designated here from syntypes: China, Hubei, Ichang [Yichang] and immediate neighbourhood, San-ya-yang, May 1888, *A.Henry* 4160 (lecto. K!) [K-000464659]; syntype: China, Hubei, Ichang,“between the mountains and the hills” received March 1886, *A.Henry* 514 (syn. K!) [K-000464658].
= Liliumbrowniivar.ferum Stapf in Elwes, Gard. Chron., ser. 3, 70: 101 (1921). Lectotype designated here from syntypes: China, Hubei, Ichang, “Nan-to and mountains northward”, February 1887, *A.Henry* 2047 (lecto. K!) [K-000464656]; syntype: China, Western Hubei, June 1907 to November 1909, *E.H.Wilson* 1447 (syn. K!) [K-000464657].

#### Holotype.

ex Japan (cultivated), Thomas Softley Ware, Hale Farm Nursery, Tottenham, London, 22 July 1885 (holo. K!) [K-000464651]. Paratype: “Hort. Ware, July 1885” (para. K!).

#### Diagnosis.

Differing from var. brownii and var. chloraster by the dark green obovate-lanceolate to oblanceolate leaves 5–7 × 1–2 cm (vs. linear to lanceolate 0.6–1 cm wide). The leaf size also decreases and becomes more sparse towards the apex of the inflorescence than in the other varieties. Corolla colour varies in the degree of colouration from finely chestnut brown markings externally to greenish.

#### Distribution.

**China**: Anhui, Fujian, Gansu, Guangxi, Guizhou, Hebei, Henan, Hubei, Hunan, Jiangsu, Jiangxi, Shaanxi, Shanxi, Sichuan, Yunnan, Zhejiang. It seems that var. viridulum does not occur in Guandong Province.

#### Ecology.

Growing along ravines on grassy slopes, in clearings of open forests and amongst low scrub, 100 to 1000 m alt. Flowering in June and July.

#### Illustration.

https://www.biodiversitylibrary.org/item/92598#page/82/mode/1up (as *Liliumjaponicum*).

## Supplementary Material

XML Treatment for Lilium brownii

XML Treatment for Lilium brownii var. brownii

XML Treatment for Lilium brownii var. chloraster

XML Treatment for Lilium brownii var. viridulum

## ﻿Unpublished sources

Kerr W (1804) Memorandum of Plants, Seeds & c. sent from China to the Royal Gardens, Kew. Conserved at Special Collections, School of Oriental and African Studies National Research Library, London.

Anon (1806) Kew Record Book (1804–1826). Library and Archives, Royal Botanic Gardens Kew, Richmond, Surrey, UK.

## References

[B1] Aiton WT (1811) *Lilium*. Hortus Kewensis; or, a catalogue of the plants cultivated in the Royal Botanic Garden at Kew Ed. 2. vol. 2. George Nicol, London, 240–241.

[B2] Anon (1832) The Horticultural Society of London, Chiswick floral exhibition. Albion and The Star 5 July 1832: e4.

[B3] Anon (1836) Obituary of Charles Brown of Slough. Windsor and Eton Express 28 May 1836: e5.

[B4] Anon (1842) Important sale of part of the superior nursery stock. The Gardeners’ Chronicle 9: e138.

[B5] Anon (1844) The Royal Botanic Society of London second Exhibition. The Gardeners’ Chronicle and Agricultural Gazette, 375 pp.

[B6] Anon (1885) Obituary of Charles Turner of The Royal Nursery, Slough. Windsor and Eton Express 16 May, 12 pp.

[B7] Anon (1843) 69^th^ Catalogue des Expositions de la Société Royale d’Agriculture et de Botanique de Gand 75^th^ Exposition. D. J. Vanderhaeghen-Hulin, Gand [Ghent].

[B8] Anon (1844) 69^th^ Catalogue des Expositions de la Société Royale d’Agriculture et de Botanique de Gand 76^th^ Exposition. D. J. Vanderhaeghen-Hulin, Gand [Ghent].

[B9] Bailey K (2019) John Reeves pioneering collector of Chinese plants and botanical art. ACC Art Books Ltd in association with the Royal Horticultural Society.

[B10] Baker JG (1871) Liliumjaponicumvar.brownii in A new Synopsis of all the known lilies IV. Gardeners’ Chronicle & Agricultural Gazette 1871 1: 708–709.

[B11] Baker JG (1875) Revision of the Genera and Species of Tulipeae. Journal of the Linnean Society. Botany 14: 211–310. 10.1111/j.1095-8339.1874.tb00314.x

[B12] Baker JG (1885) Liliumbrowniivar.viridulum Baker. The Gardeners’ Chronicle [n.s.] 24: e134.

[B13] Bentham G (1835) Report on the Exhibitions of the Horticultural Society. Transactions of the Horticultural Society of London ser. 21: e534.

[B14] Brookes S (1822) Notice relative to the flowering of *Liliumjaponicum*. In a letter to the Secretary. Read August 7^th^ 1821. Transactions of the Horticultural Society 4: 551–553. [W. Nicol, London]

[B15] Brown E (1864) Testimonial to Mr. Ingram Chief Gardener to Her Majesty at Frogmore. The Gardeners’ Chroonicle and Agricultural Gazette, 1129 pp.

[B16] Brown T Brown E (1841) announcement in The Gardeners’ Chronicle 47: e762.

[B17] Brown T (1842) The Salt Hill Grand Dahlia Show. The Gardeners’ Chronicle 37: e601.

[B18] Brown T (1843) The Salt Hill Grand Dahlia Show. The Gardeners’ Chronicle 37: e641.

[B19] Brown T (1844) The Salt Hill Grand Dahlia Show. The Gardeners’ Chronicle and Agricultural Gazette, 641 pp.

[B20] Bury PS (1831) *Liliumjaponicum*. A selection of hexandrian plants belonging to the natural orders Amaryllidae and Liliaceae: species 49 t. 2. Robert Havell, London. 10.5962/bhl.title.539

[B21] Cannart d’Hamale F (1870) Monographie historique et littéraire de Lis. T. Ryckmans-Van Deuren. Malines [Mechelin], Belgium.

[B22] Cavalerie J (1911) Les Liliacées de Kouy-Tchéou. Bulletin de géographie botanique 21: 243–248.

[B23] Clarke SR (1822) Hortus Anglicus; or, the modern English garden. vol. 1: 332. F. C. & J. Rivington, London.

[B24] Compton JA (2015) *Wisteriasinensis*, on the slow boat from China: The journey of *Wisteria* to England (Leguminosae) Fabaceae. Curtis’s Botanical Magazine 32(3–4): 248–293. 10.1111/curt.12112

[B25] Dean R (1885) The Royal Nurseries Slough and Charles Turner. The Gardeners Magazine 28: e264.

[B26] Demeunynck Devaux [Eds] (1842) Annuaire Statistique du Département du Nord 14^th^ Année. L. Danel, Lille; Vanackere fils, Lille.

[B27] Du Y-P He H-B Wang Z-X Li S Wie C Yuan X-N Cui Q Jia G-X (2014) Molecular phylogeny and genetic variation in the genus *Lilium* native to China based on the internal transcribed spacer sequences of nuclear ribosomal DNA. Journal of Plant Research 127(2): 249–263. 10.1007/s10265-013-0600-4 24212402

[B28] Duchartre PES (1870) Notes et Mémoires III. Observations sur le genre Lis (*Lilium* Tourn.) apropos du catalogue de la collection de ces plantes qui a été formée par M. Max Leichtlin de Carlsruhe. Journal de la Société Impériale et centrale d’horticulture de France ser. 2(4): 341–359.

[B29] Dumont de Courset GLM (1814) *Lilium*. Le botaniste cultivateur ed. 2, tome 7 Supplément: 54–55. Deterville, Paris; Goujon, Paris.

[B30] Elliott B (2001) Flower Shows in Ninteenth-Century England. Garden History 29(2): 171–184. 10.2307/1587369

[B31] Elliott B (2004) The Royal Horticultural. Social History: 1804–2004. [Phillimore & Co Ltd. Sussex in association with the Royal Horticultural Society, London.]

[B32] Elwes HJ (1877) *Liliumbrownii*. A monograph of the genus *Lilium* part 3: t. 8. Taylor & Francis, London.

[B33] Elwes HJ (1921) Two little known lilies. The Gardeners’ Chronicle: a weekly illustrated journal of horticulture and allied subjects ser 3. 69: 100–101.

[B34] Fan F (2003) Science in a Chinese Entrepôt: British Naturalists and their Chinese Associates in Old Canton. Osiris ser. 218: 60–78. 10.1086/649377

[B35] Franchet AR (1892) Les Lis de la Chine et du Thibet dans l'herbier du Muséum de Paris. Journal de Botanique (Morot) 6(18): 304–321.

[B36] Fraser Maxwell [Dorothy May] (1973) The History of Slough. Slough Corporation, 99–100.

[B37] Gao Y-D Gao X-F (2014) Taxonomic notes on Chinese *Lilium* L. (Liliaceae) with proposal of three nomenclatural revisions. Phytotaxa 172(2): 101–108. 10.11646/phytotaxa.172.2.5

[B38] Gao Y-D Harris AJ He X-J (2015) Morphological and ecological divergence of *Lilium* and *Nomocharis* within the Hengduan Mountains and Qinghai-Tibetan Plateau may result from habitat and specialization and hybridization. BMC Evolutionary Biology 15(1): e147. 10.1186/s12862-015-0405-2 PMC4518642 26219287

[B39] Givnish TJ Skinner MW Rešetnik I Ikinci N Kriebel R Lemmon AR Lemmon EM Gao Y-D (2020) Evolution, Geographical Spread and Floral Diversification of the Genus *Lilium* with special reference to the lilies of North America. Evolution 74(3): 26–44.

[B40] Glenny G (1834) Metropolitan Society shows. Royal Lady’s Magazine [n.s] 3: e28.

[B41] Glenny G (1845) Unrivalled collection of Tulips. The Gardeners’ Chronicle and Agricultural Gazette 26 April, 282.

[B42] Goodman J Jarvis C (2017) The John Bradby Blake drawings in the Natural History Museum, London: Joseph Banks puts them to work. Curtis’s Botanical Magazine 34(4): 251–275. 10.1111/curt.12203

[B43] Hardy HC (1811) A Register of Ships, employed in the service of the Honourable East India Company from the year 1760 to 1810. Black, Parry & Kingsbury, London.

[B44] Hardy HC (1820) A Register of Ships, employed in the service of the Honourable East India Company from the year 1760 to 1819. Black, Kingsbury, Parbury & Allen, London.

[B45] Hayashi K (2016) *Lilium* L. In: Iwatsuki K Boufford DE Ohba H (Eds) Flora of Japan IVb AngiospermaeMonocotyledoneae (b).Kodansha, Tokyo, 110–117.

[B46] Hooker JD (1892) *Lilium*. The Flora of British India vol. 6. L. Reeve & Co., London, 349–352.

[B47] Houttuyn M (1780) Natuurlyke Historie of uitvoerige beschryving der dieren, planten en mineralen volgens het samenstel van den heer Linnaeus met naauwkeurige afbeeldingen vol. 2 pt 12. De Erven van F. Houttuyn, Amsterdam.

[B48] Huang J Yang L-Q Yu Y Liu Y-M Xie D-F Li J He X-J Zhou S-D (2018) Molecular phylogenetics and historical biogeography of the tribe Lilieae (Liliaceae): Bi-directional dispersal between biodiversity hotspots in Eurasia. Annals of Botany 122: 1245–1262. 10.1093/aob/mcy138 30084909 PMC6324749

[B49] Ker-Gawler JB (1813) *Liliumjaponicum* in Curtis’s Botanical Magazine 38: t. 1591.

[B50] Kilpatrick J (2007) Gifts from the Gardens of China. Frances Lincoln Ltd. London.

[B51] Krelage JH (1878) *Liliumbrownii* v. *L.japonicumcolchesteri*, The Garden 13.

[B52] Lee CS Kim S-C Yeau SH Lee NS (2011) Major Lineages of the Genus *Lilium* (Liliaceae) Based on NrDNA ITS Sequences, with Special Emphasis on the Korean Species. Journal of Plant Biology 54(3): 159–171. 10.1007/s12374-011-9152-0

[B53] Lemaire C (1845) *Liliumbrownii*. Flore des Serres et des Jardins de l’Europe 1: 257–259. [t. *Liliumbrownii*.]

[B54] Lemaire C (1848) Lis de Brown, *Liliumbrownii*. Herbier Général de l'Amateur ser. 2 5: 74–76. [t. *Liliumbrownii*.]

[B55] Lemoinier A (1841) Exposition d’été. Annales de la Société d'Horticulture du Département du Nord 13: 3–11.

[B56] Lemoinier A (1842) Société d’Horticulture du Département du Nord. Secrétaire-adjoint M. Lemoinier fils. Annuaire Statistique du Département du Nord. 14^th^ année: 379. L. Danel Lille; Vanackere fils, Lille.

[B57] Levéillé AAH (1909) Liliumlongiflorumvar.purpureoviolaceum H.Lév. Repertorium Specierum Novarum Regni Vegetabilis 6: 264.

[B58] Liang S-Y Tamura MN (2008) *Lilium*. In: Wu Z, Raven PH, Hong D (Eds) Flora of China 24: 135–149.

[B59] Livingstone J (1819) Dr. Livingstone’s Letter to the Horticultural Society of London. Indo-Chinese Gleaner 9 (July): 126–131.

[B60] Livingstone J (1822) On the Transportation of plants from China to England. Transactions of the Horticultural Society of London 3: 421–429.

[B61] Loddiges G (1820) *Liliumjaponicum*. Loddiges Botanical Cabinet 5: Tab. 438.

[B62] Loiseleur-Deslongchamps JLA (1822) *Liliumjaponicum*. Herbier Général de l’Amateur 6: t. 375. Audot, Paris.

[B63] Loudon JC (1831) Florists guide number XL for October. The Gardener’s Magazine and register of domestic and rural improvement vol. 7.

[B64] Loudon JC (1833) Art. VII. London Horticultural Society and Garden. The Gardener’s Magazine and register of domestic and rural improvement vol. 9.

[B65] Loudon JC (1837) Art VII. London Horticultural Society and Garden, from the garden of the Society July 18 1837. The Gardener’s Magazine and register of domestic and rural improvement [n.s.] vol. 3.

[B66] Loudon JC (1838) Proceedings of the Horticultural Society of London. The Gardener’s Magazine and Register of rural and domestic improvement [n.s.] vol. 4.

[B67] Marnock R (1841) Chiswick Horticultural Show July 4. Floricultural Magazine and Miscellany of Gardening 5: 60–62.

[B68] Morren C (1847) Note 1 (on *Liliumbrownii*). Annales de la Société royale d’agriculture et de botanique de Gand 3: e309.

[B69] Nishikawa T Okazaki K Arakawa K Nagamine T (2001) Phylogenetic analysis of section Sinomartagon in genus *Lilium* using sequences of the Internal Transcribed Spacer region in nuclear ribosomal DNA. Breeding Science 51(1): 39–46. 10.1270/jsbbs.51.39

[B70] Okubo H Hiramatsu M Masuda J-I Sakazono S (2012) New insight into Liliumbrowniivar.colchesteri. Floriculture and Ornamental Biotechnology 6 (Special Issue 2): 44–52.

[B71] Paxton J (1834) Article II. London Horticultural Society. The Horticultural register, and general magazine: 381–383.

[B72] Pépin PD (1847) Igname et *Liliumbrownii* présénts à la Société d'horticulture. Revue Horticole ser. 31: e345.

[B73] Planchon JE (1854) *Liliumodorum*. Flore des serres et des jardins de l’Europe. 9: 53 t. 876.

[B74] Poiret JLM (1813) *Liliumjaponicum*. Encyclopédie méthodique, Botanique, Supplement 3. H. Agasse, Paris.

[B75] Poiret JLM (1823) *Liliumjaponicum*. Dictionnaire des Sciences Naturelles 27. F. G. Levrault, Strasbourg; le Prince, Paris, Le Normant, Paris.

[B76] Poiteau PA (1843) Autre rectification. Revue Horticole 5(30): e406.

[B77] Poiteau PA (1844) Autres plantes nouvelles ou peu connues. Revue Horticole 5(34): 495–496.

[B78] Regel E (1854) Neue Zierpflanzen 2. Gartenflora 3: 234–238. 10.1007/BF02212352

[B79] Rivers T (1838) Review. The Rose Amateur’s Guide. The Floricultural Cabinet and Florist’s Magazine 6: 17–19.

[B80] Robinson W (1886) Obituary of Thomas Brown. The Garden: an illustrated weekly journal of gardening in all its branches 30: e598.

[B81] Slater I (1844) Pigot & Co’s Royal National and Commercial Directory and Topography for the counties of Berkshire etc. I. Slater, London.

[B82] Spae D (1845) *Liliumbrownii*. Annales de la Société Royale d’agriculture et de Botanique de Gand 1: 437–438, t. 41.

[B83] Spae D (1847) Mémoire sur les espèces du genre Lis, Mémoires Couronnés et Mémoires des Savants Étrangers, publiés par L’Académie Royale de sciences, des lettres et des beaux arts de Belgique 19(5): 1–46.

[B84] Stearn WT (1948) The Botanical Names of some lilies. Gardeners’. The Chronicle 1948: 4–5.

[B85] Synge PM (1980) Lilies, a Revision of Elwes’ Monograph of the genus *Lilium* and its Supplements. Batsford Ltd., London.

[B86] Thunberg CP (1784) Flora Japonica sistens plantas insularum Japonicarum. J. G. Müller, Lipsia [Leipzig]. 10.5962/bhl.title.120525

[B87] Turland NJ Wiersema JH Barrie FR Greuter W Hawksworth DL Herendeen PS Knapp S Kusber W-H Li D-Z Marhold K May TW McNeill J Monro AM Prado J Price MJ Smith GF (Eds) (2018) International Code of Nomenclature for algae, fungi, and plants (Shenzhen Code) adopted by the Nineteenth International Botanical Congress Shenzhen, China, July 2018. Regnum Vegetabile 159. Koeltz Botanical Books, Glashütten. 10.12705/Code.2018

[B88] Van Eeden AC (1876) *Liliumbrownii*. Album van Eeden. Haarlem’s Flora afbeeldingen in kleurendruk van verschillende bol-en knolgewassen. A. C. van Eeden & Co. Haarlem.

[B89] Van Houtte LB (1833) *Liliumlongiflorum*. Horticulteur Belge, journal des jardiniers et amateurs 1.

[B90] Van Houtte LB (1845) *Liliumbrownii*, Hortus vanhoutteanus ou description de plantes Nouvelles, rare sou peu connus. 1. F. & E. Gyselynck, Gand [Ghent].

[B91] Wallace A (1875) *Liliumjaponicumcolchesteri*. The Garden 8.

[B92] Wallace A (1878) *L.brownii* v. *L.japonicumcolchesteri*. The Garden 13.

[B93] Wallace RW (1932) Hybrid lilies. Royal Horticultural Society Lily Year Book 1: 42–52.

[B94] Wilson EH (1925) The Lilies of Eastern Asia. Dulau & Co. Ltd., London.

[B95] Woodcock HBD Stearn WT (1950) Lilies of the World, their Cultivation and Classification. Country Life Ltd., London; Charles Scribner’s Sons, New York.

